# Targeting the PD-1 pathway in patients with relapsed classic Hodgkin lymphoma following allogeneic stem cell transplant is safe and effective

**DOI:** 10.18632/oncotarget.7177

**Published:** 2016-02-03

**Authors:** Jose Caetano Villasboas, Stephen M. Ansell, Thomas E. Witzig

**Affiliations:** ^1^ Mayo Clinic, Rochester, MN, USA

**Keywords:** Hodgkin lymphoma, immunotherapy, PD-1 inhibitors, allogeneic stem cell transplantation, pembrolizumab

## Abstract

Patients with classic Hodgkin lymphoma (cHL) that has relapsed after autologous or allogeneic transplant have limited treatment options and a poor prognosis. Immunotherapy with agents that target the PROGRAMMED DEATH 1 (PD-1) receptor have demonstrated clinical activity with durable responses in early-phase clinical trials in this patient population; however, patients with a history of allogeneic stem cell transplantation (SCT) were intentionally excluded from participation in those studies due to concerns for reactivation of graft-versus-host disease (GVHD). We describe the clinical course of two patients with advanced cHL and prior treatment with allogeneic stem cell transplantation (SCT) that were treated with the PD-1 inhibitor pembrolizumab. Both patients had no active graft-versus-host disease (GVHD) at the time initiation of therapy and were maintained on low-dose prednisone. Treatment with pembrolizumab was well tolerated and not associated with reactivation of GVHD. Both patients responded (1 partial, 1 complete) and remain on therapy as of November 30, 2015. This report indicates that immunotherapy targeting the PD-1 pathway can be safely administered to patients with cHL and a history of allogeneic SCT and produce tumor responses. Further studies in this patient population are needed.

## INTRODUCTION

The safety and therapeutic activity of monoclonal antibodies targeting the PROGRAMMED DEATH 1 (PD-1) receptor in advanced classic Hodgkin lymphoma (cHL) has been demonstrated in two recent publications [1, 2]. Both studies excluded patients with a previous history of allogeneic bone marrow transplantation, primarily due to safety concerns related to graft-versus-host disease (GVHD). As allogeneic stem cell transplantation (SCT) remains a viable option for the treatment of select younger patients with advanced cHL [3, 4], these patients are faced with limited treatment options if they fail allogeneic transplant. Herein we describe the clinical course of two patients with relapsed cHL treated off-label with the PD-1 inhibitor pembrolizumab who had previously undergone allogeneic SCT.

## CASE REPORTS

### Patient 1

**Figure 1:**
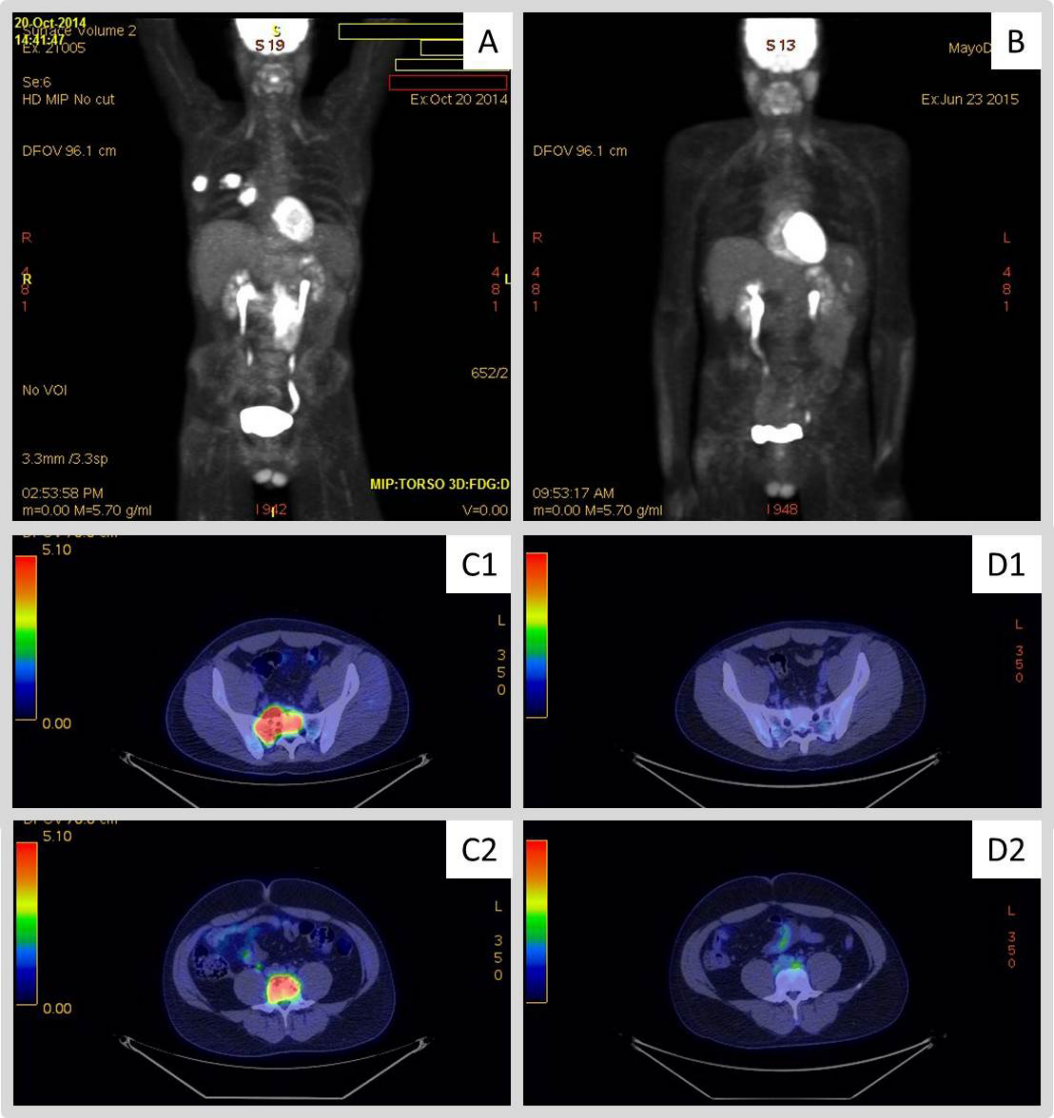
18F-fluorodeoxy-glucose–positron-emission tomography (FDG-PET) images obtained to evaluate response to therapy with pembrolizumab Panels **A.** and (C1/C2) display pre-treatment scans for patient 1 and 2, respectively. Hypermetabolic masses are observed in the abdomen, lateral right chest wall soft tissue extending into the lung parenchyma, and right hilum on panel (A). Panel (C1) displays hypermetabolic skeletal lesion involving upper right sacrum while (C2) demonstrates involvement of L4. Panels (B) and (D1/D2) display follow-up scans obtained to evaluate response to therapy in patient 1 and 2, respectively. Panel **B.** displays PET-CT scan obtained on patient 1 after 3 cycles of pembrolizumab showing no evidence of FDG-avid malignancy. Panel (D1 and D2) displays PET-CT scan obtained on patient 2 after 4 cycles of pembrolizumab showing resolution of FDG-avid bone lesions.

A 30-year-old man was diagnosed with stage IIA cHL in 2007 and treated with standard ABVD (doxorubicin, bleomycin, vinblastine and dacarbazine) for 6 cycles. No evidence of FDG-avid malignancy was identifiable on PET-CT obtained after 2 cycles. He proceeded to receive adjuvant radiotherapy to the right neck and chest area. His disease relapsed 6 months later and he was treated with salvage ICE (ifosfamide, carboplatin and etoposide) followed by autologous SCT in March of 2008. Less than six months after autologous SCT the patient was found to have biopsy-proven relapsed disease in the lungs. He was treated with GVD (gemcitabine, vinorelbine and pegylated liposomal doxorubicin) and underwent nonmyeloablative allogeneic SCT from a matched related donor in January of 2009. His post-transplant course was complicated by acute GVHD involving the skin and gut treated with corticosteroids. He underwent a mediastinoscopy in August 2009 to investigate mediastinal lymphadenopathy and was found to have relapsed disease. Withdrawal of immunosuppression was attempted; however, follow-up imaging demonstrated progressive disease in the fall of 2009. At that time, grade 1 GVHD of the liver was found on biopsy done to investigate elevated transaminases. He arrived at our institution in November of 2009 for a second opinion and was treated with single-agent everolimus as part of a clinical trial (NCT01022996) [5]. After 18 months on therapy with stable disease the patient developed progression. By October 2014 the patient had received multiple lines of therapy including the histone deacetylase (HDAC) inhibitor panobinostat, single-agent brentuximab vedotin, single-agent bendamustine, weekly single-agent vinblastine, cyclophosphamide plus carmustine, ICE, and everolimus plus lenalidomide. Unfortunately, his disease continued to relentlessly progress despite these therapies. By October 2014 the patient had exhausted all reasonable therapies and the results of phase I trials of PD-1 inhibitors for relapsed cHL were published. Unfortunately, his prior history of allogeneic SCT rendered him ineligible for participation in any of PD-1 inhibitor studies. After counseling the patient as to potential risks and benefits, he agreed to off-study, off-label therapy with pembrolizumab. On October 21, 2014 the patient received his first infusion of pembrolizumab (2 mg/kg/dose repeated every 3 weeks). At the time of initiation of therapy he had no evidence of active GVHD on a stable dose of 2.5 mg of prednisone daily (Table 1). On January 2015 (following 3 cycles of pembrolizumab) an 18F-fluorodeoxy-glucose–positron-emission tomography (FDG-PET) showed interval resolution of the FDG-avid right lung mass, hilar lymphadenopathy, chest wall nodule and intra-abdominal masses consistent with a complete response (Figure 1). This was reconfirmed after 9 cycles. As of November 2015, the patient had received 16 doses with a durable response and no reactivation of GVHD.

**Table 1:** Clinical characteristics of the two patients with advanced cHL and history of allogeneic stem cell transplant treated with pembrolizumab

PRE-TREATMENT	PATIENT 1	PATIENT 2
Age (years)	30	30
Gender	Male	Male
Year of diagnosis	2007	2008
Autologous SCT	March 2008	April 2009
Allogeneic SCT	January 2009	January 2014
Number of other prior systemic therapies	11	8
Previous brentuximab vedotin	Yes	Yes
Chronic GVHD (location; stage)*	Liver (score 1)	None
Prednisone dosage	2.5 mg daily	2.5 mg daily
Date of first pembrolizumab infusion	October 21, 2014	July 7, 2015
POST-TREATMENT	PATIENT 1	PATIENT 2
Best overall response	Complete response	Partial Response
Chronic GVHD (location; stage)*	Liver (score 1)	None
Date of last infusion	October 26, 2015	October 27, 2015
Number of total infusions	16	7
Date of last radiological assessment	June 23, 2015	October 5, 2015
Treatment status	Ongoing	Ongoing

Top panel displays pre-treatment variables. Bottom panel displays follow-up information after initiation of therapy with pembrolizumab.

Abbreviations: SCT, stem cell transplantation; GVHD, graft verses host disease

* According to National Institutes of Health Consensus Development Project on Criteria for Clinical Trials in Chronic Graft-versus-Host Disease [12].

### Patient 2

The second patient is a 30-year-old man who was diagnosed with stage IIIS cHL in 2008. His disease was initially treated with first-line ABVD followed by adjuvant radiotherapy. He relapsed less than a month after the end of radiotherapy. He was subsequently treated with salvage ICE and progressed after 2 cycles. Therapy with GVD followed and he later underwent autologous SCT in April 2009 with adjuvant radiotherapy. In April 2013 the patient developed biopsy-proven recurrent disease that was treated with brentuximab vedotin followed by GVD with good response. The patient underwent nonmyeloablative allogeneic SCT from a matched related donor in January 2014 without complications or evidence of GVHD. In July 2014 the patient developed biopsy-proven relapsed disease in the axial skeleton with significant pain. Subsequent treatment regimens were ineffective and included single-agent brentuximab vedotin and on-study with panobinostat and everolimus. Similar to patient 1, by July 7, 2015 the patient had exhausted all reasonable therapies and consented to off-study pembrolizumab (2 mg/kg/dose repeated every 3 weeks). At the time of initiation of therapy he had no evidence of active GVHD and was maintained at the dose of 2.5 mg of prednisone daily (Table 1). After the second infusion of pembrolizumab the patient noted significant improvement in pain secondary to bone metastases and discontinued the use of analgesics. On October 5, 2015 – following 4 cycles of pembrolizumab – a PET-CT showed resolution of all FDG-avid bone lesions with persistent uptake in spleen and several small abdominal nodes consistent with partial response (Figure 1). As of November 2015, he continues pembrolizumab therapy (total of 7 infusions to date) without exacerbation of GHVD.

## DISCUSSION

We have described the successful clinical course of two patients with relapsed refractory cHL treated with pembrolizumab after having failed multiple lines of therapy including allogeneic SCT. Both of our patients responded to therapy (1 CR and 1 PR) and remain on treatment without evidence of disease progression. Treatment with pembrolizumab in these two patients was not associated with reactivation of GVHD.

The current report is the first to describe the safe and successful use of pembrolizumab in patients with cHL who had previously been treated with allogeneic SCT. Although the PD-1 pathway has been implicated as an escape mechanism for malignant stem cells following allogeneic transplant [6], data on the safety and efficacy of immune checkpoint inhibitors in this setting are scarce. In animal models, treatment with PD-1 inhibitors is associated with augmentation of a xenograft-versus-host disease in mice transferred with human lymphocytes [7]. Other types of immune checkpoint inhibitors such as ipilimumab [8] and pidilizumab [9] have been tested in early phase clinical trials and did include patients with a previous history of allogeneic SCT. A case report describing the use of immunotherapy for the treatment of metastatic melanoma in long-term survivors of allogeneic SCT has recently been published [10]. With regards to relapsed HL, there has been a report of one case of relapsed HL after allogeneic SCT that was successfully treated with nivolumab [11]. In all these cases, treatment with immune checkpoint inhibitors did not seem to contribute to exacerbation or reactivation of GVHD.

Our limited experience cannot necessarily be extrapolated to all patients who present with relapsed cHL after having failed previous treatment with allogeneic SCT. It is important to note that both of our patients had no evidence of active GVHD at the time of therapy with pembrolizumab and were maintained a low-dose prednisone regimen. This may have contributed to safe administration of this agent. These encouraging results with respect to safety and efficacy in this important group of patients should spur additional testing of PD-1 inhibitors in this patient population.
